# Blood biochemical parameters and organ development of brown layers fed reduced dietary protein levels in two rearing systems

**DOI:** 10.5713/ab.21.0145

**Published:** 2021-06-24

**Authors:** Eduardo de Faria Viana, Heloisa Helena de Carvalho Mello, Fabyola Barros Carvalho, Marcos Barcellos Café, Nadja Susana Mogyca Leandro, Emmanuel Arnhold, José Henrique Stringhini

**Affiliations:** 1 Federal Institute Goiano, GO, 75790-000, Brazil; 2 Department of Animal Science, Federal University of Goiás, GO, 74690-900, Brazil

**Keywords:** Blood Plasma, Digestive System, Poultry Housing, Protein Content, Welfare

## Abstract

**Objective:**

An experiment was conducted to evaluate the effect of different levels of crude protein (CP) and two rearing systems (cage and floor), on blood parameters and digestive and reproductive organ development of brown laying hens.

**Methods:**

A total of 400 Hisex Brown laying hens between 30 and 45 weeks of age were distributed in a completely randomized design and a 2×4 factorial arrangement, with main effects including two rearing systems (cage and floor) and levels of CP (140, 150, 160, and 180 g/kg), in a total of eight treatments and five replicates of 10 birds each with initial body weight of 1,877 g (laying hen in cage) and 1,866 g (laying hens in floor). The parameters evaluated were plasma total protein, albumin, uric acid, total cholesterol, relative weights of oviduct, abdominal fat, liver, gizzard, crest and dewlap, length of small intestine and oviduct.

**Results:**

The blood parameters were similar in birds reared in cage and floor systems. The birds reared on the floor showed greater small intestine and oviduct weight (%) and lower liver and pancreas weight (%). A significant interaction was observed between factors for the relative gizzard, crest and dewlap weight, serum protein, uric acid, and total cholesterol (p<0.05). The diets with 140 g/kg CP resulted in lower serum protein and lower cholesterol in birds reared in floor system, while birds reared in cage system showed no effect of CP on both parameters. Birds reared in cage and fed with 140 and 150 g/kg CP presented lower uric acid. The group of birds reared in floor system fed 180 g/kg had greater uric acid.

**Conclusion:**

The dietary protein level can be reduced up to 140 g/kg for Hisex Brown hens (30 to 45 weeks of age) without an important effect on metabolic profile and organ development in both rearing systems.

## INTRODUCTION

The rearing systems that allow laying hens to express their natural behaviors have been used to improve the hens welfare. Many countries have banned the use of conventional cages in laying hen production. The benefits of furnished and open systems are reduction of the prevalence of keel-bone fractures [1] and reduction of fearfulness [2]. Furthermore, the improvement of egg production and better egg internal and external quality [3,4] has been related as the benefits of open systems, like a floor system. The eggs produced in conventional cages compared with those laid in free-range farming conditions, presented higher concentrations of total lipids, cholesterol, and gross energy [5]. It has been reported that eggs produced by birds in open systems present a higher yolk percentage compared to cage system [3,6], and that the egg mineral content is affect by rearing system as well [7]. In this way, egg composition is affected by the system, therefore the blood parameters of laying hens that are related to egg formation could be affect too. Reducing space allowance in or eliminating access to a nest box results in disruption of biological function [8]. Based on this, the information about the metabolic and organ development effects in laying hens raised in alternative systems must be improved.

In addition to replacement of rearing system, poultry diets have been formulated based on the use of digestible amino acids, considering the ideal protein concept. It is reported that the reduction of dietary protein from 17% to 15.7% with amino acid supplementation is enough to maintain performance of Brown laying hens [9]. The rearing system affects both the performance of hens and the egg quality characteristics [10], that are associated to crude protein (CP) requirements by birds. Therefore, the reduction of dietary protein should be studied in different rearing systems, to determine the rearing system affect the nutritional requirements of birds. According to de Almeida Brainer et al [11] hens housed in free-range systems have a greater CP requirement for maintenance than the CP requirement for production, due to its lower productive potential and greater caloric expenditure, given the physical activities that occur under semi-intensive management.

Blood biochemical parameters had been used as metabolic variables to assess the welfare of poultry [12]. The behavior, blood corticosterone and blood biochemical parameters in laying hens change during the stress [13,14]. The blood biochemical parameters provide information about immune system of birds, which is associated with environmental conditions, and can aid in the assessment of rearing systems. In addition, the digestive and reproductive organs development, and sexual characteristics (crest and dewlap) can be used to assess the birds’ metabolism and development.

The biochemical composition of the blood plasma reflects the metabolic situation of the tissues, making it possible to evaluate changes in the organ’s functions and the adaptation of the animal to nutritional and physiological challenges, in some cases, imposed by stressful situations [15]. Hens reared in floor systems are more challenged by microorganisms than those reared in cage systems. A trend toward increased bird-to-bird transmission of *Salmonella* enteridis was detected in the aviary and floor system compared with the cage systems [16]. In fact, Parisi et al [17] showed that free-range eggs had higher prevalence of *Salmonella* and *Campylobacter* spp. than battery cage eggs, indicating a possible challenge to hens reared in floor system. In this context, the dietary protein requirements could be different in hens under different housing conditions as the protein participates of immune system in animals.

Due to these factors and considering that less is understood about the protein requirement for laying hens reared in a floor system, we hypothesized that the replacement from conventional cages to floor can change the protein requirements of the birds. In this context, it is important to undertake research focused on the serum biochemistryl and organ development of laying hens in floor and cage systems fed different dietary CP.

This study was conducted to investigate the blood biochemical parameters, the digestive and reproductive organs development of Hisex Brown layers, from 30th to 45th weeks of age in two rearing systems, receiving reduced dietary protein.

## MATERIALS AND METHODS

### Animal care and study site

This experiment was performed in accordance with Ethics Committee on the Use of Animals (CEUA/UFG), under protocol 312/11. The experiment was carried out in the city of Urutaí in the state of Goiás, Brazil, Latitude: 17° 27′ 49″ S, Longitude: 48° 12′ 06″ W, Altitude: 807 m.

### Animals, experimental design, and management

Four hundred Hisex Brown layers, from 30 to 45 weeks of age, were allotted in a completely randomized design and a 2×4 factorial arrangement, with main effects including two rearing systems (cage and floor) and four levels of CP (140, 150, 160, and 180 g/kg CP), with five replicates of ten birds each with initial body weight of 1,877 g (laying hen in cage) and 1,866 g (laying hens in floor).

The experimental diets were isonutritive and formulated on the ideal protein concept, according to Rostagno et al [18] (Table 1).

The floor rearing system consisted of 20 boxes with rice hulls as a litter material. Each box was equipped with a pendular drinker, a linear tube feeder and a nest. The boxes had a measurement of 2.2×1.5×3 m (length×width×height) and the floor litter had a height of 10 cm. The nest, built out of wood, had three holes (33×40×45 cm) and an elevation of 10 cm in relation to the litter line. The density of 3.3 m^2^/bird was adopted on the floor system. The conventional cages size was 100×37×40 cm, with four divisions of 25 cm. The density of 500 cm^2^/bird in the cages was adopted. During the entire experimental period, *ad libitum* feed and water were provided and a lighting program with 16 h light and 8 h dark period, as indicated in the Hisex Brown Management Manual. The temperature inside the poultry houses were controlled with a maximum-minimum thermometer (Incoterm, Urutaí, GO, Brazil). The average of minimum and maximum temperature of cage system were 22.3°C and 30.1°C, respectively. The average of minimum and maximum temperature of floor system were 22.5°C and 29.9°C, respectively.

### Blood sampling

The blood parameters analysis was performed on the 2nd and 4th periods of egg production (37th and 45th weeks of age) and results were presented as mean of two periods.

After 6 h fasting, five hens from each treatment (one of each replication) at 37th and 45th weeks of age, totalizing ten birds per treatment, were selected to measure the blood parameters. About 5 mL of blood was collected from cardiac puncture, in tubes containing anticoagulant (sodium fluoride). The plasma was separated by centrifugation. The levels of plasmatic total protein, albumin, uric acid, and total cholesterol were determined on an automated biochemical analyzer.

### Organ properties

After collecting blood, one bird per repetition was euthanized by cervical dislocation. The small intestine, liver, pancreas, gizzard, crest, dewlap, oviduct, and abdominal fat were collected and expressed in terms of relative weight per 100 g of body weight. The length of small intestine and oviduct were measured. The birds were weighed on a precision scale. The measurement of the length of the intestines was performed from the initial portion of the duodenum to the rectum, and that of the oviduct, from the infundibulum to the vagina.

### Statistical analysis

The experiment was designed as a 2×4 factorial arrangement and the data were analyzed using the R program (version 2020) in the model as follows:


$${\text{Y}}_{\text{ijk}}=\mu +{\text{a}}_{\text{i}}+{\text{b}}_{\text{j}}+{\left(\text{ab}\right)}_{\text{ij}}+{ɛ}_{\text{ijk}},$$

in which y_ijk_ = value observed in the rearing system i (i = 1, 2), level j (j = 1, 2, 3, 4), and repetition k (k = 1, 2, 3, …, 5); μ = overall mean of the experiment; a_i_ = fixed effect of the system i (i = 1,2); b_j_ = fixed effect of the level j (j = 1, 2, 3, 4); (ab)_ij_ = fixed effect of the interaction between system i (i = 1, 2) and level j (j = 1, 2, 3, 4); and ɛ_ijk_ = random error in the system i (i = 1, 2), level j (j = 1, 2, 3, 4), and repetition k (k = 1, 2, 3, …, 5).

Data were submitted to analysis of variance. The significance of difference among the treatments was assessed using the Scott Knott test. The significance adopted was p-value <0.05.

## RESULTS

### The effect of rearing system

The small intestine length was not affected by the rearing system (Table 2). Compared to cage system, birds reared on the floor system showed greater small intestine (p = 0.0223, Table 2), greater oviduct (p<0.001, Table 3) and lower liver (p<0.001, Table 2) and pancreas (p = 0.0082) relative weights (Table 2). The oviduct length, the percentage of abdominal fat, the relative weight of crest and dewlap were not affected by the rearing system (Table 3). The blood parameters were similar in birds reared in cage and floor system (Table 4).

### The effect of crude protein content

The small intestine length was negatively affected using 140 and 180 g/kg of CP (p = 0.0127, Table 2). Relative weight of small intestine was lower in birds fed 150 and 180 g/kg of CP (p = 0.0049, Table 2). The CP did not affect the relative weight of liver, and pancreas (Table 2), oviduct length, relative weight of oviduct (Table 3) and serum albumin content (Table 4).

### The statistical interaction effect for the studied factors

There were significant interactions between protein levels and raising system for the relative weight of gizzard (p = 0.0386, Table 2), crest (p = 0.0481, Table 3) and dewlap (p = 0.0067, Table 3) and to serum protein (p = 0.0138, Table 4), uric acid (p<0.001, Table 4) and total cholesterol (p = 0.0094, Table 4). Birds reared in floor system and fed diets with 160 and 180 g/kg CP presents lower relative gizzard weight, but birds reared in cage system showed no differences for this variable. The diet containing 140 g/kg CP resulted in the highest relative crest weight on birds reared in cage system. Birds reared in floor system and fed diets with 140 and 150 g/kg CP presented greater relative crest weight compared to other groups. For dewlap relative weight, birds on cage system showed greater values when fed diets with 140 and 160 g/kg CP, and hens raised in floor had showed greater relative with diets containing 160 and 180 g/kg CP.

In observation of the data presented in Table 4, the birds fed diets containing 140 g/kg CP showed lower serum protein and lower total cholesterol when reared in floor system, while the ones reared in cage system had no effect of CP on both parameters. Birds reared in cage system that were fed diets with 140 and 150 g/kg CP presented lower uric acid when compared to birds fed 160 and 180 g/kg CP rations. The group of birds reared in floor system fed 180 g/kg CP had greater uric acid, compared to the hens that received 160 g/kg CP and the lowest values were observed for the ones fed 140 and 150 g/kg CP.

## DISCUSSION

The changing of rearing system from cage to floor system is a trend aimed at improving hens’ welfare. In the present study verified that laying hens reared in floor system did not show any difference in blood parameters compared to those birds reared in cage system. Blood parameters provide information on a bird’s metabolism, stress response and consequently indicate effects that can affect the performance results. It was confirmed that stress can cause elevations in blood levels of corticosterone, glucose, cholesterol, and high density lipoprotein, whereas triglycerides levels were reduced by adrenocorticotrophic hormone (ACTH) treatment [19]. Negative correlations between cholesterol content in serum and egg can indicate that eggs from hens reared in cages contained significantly more cholesterol than eggs laid by hens reared in floor pens [20]. In this context, the manipulation of serum cholesterol could result in different content in the eggs. The housing system affects the concentration of cholesterol where lower values were found on litter in comparation to cage system [21]. However, in the present experiment, the total cholesterol was similar between the rearing systems (cage and floor).

The serum content of protein, albumin, uric acid, and cholesterol are affected by age [22], pathological factors [23] and dietary additives [24]. In this experiment, a significant interaction between the dietary CP and rearing system on serum protein, uric acid, and total cholesterol of hens was observed. Exception made for albumin serum levels; the other blood parameters were affected by diets when birds were reared in floor system. On the other hand, only serum uric acid was affected by the dietary CP in birds housed in cage system. A significant interaction between the factors corresponds with the findings from Kraus et al [21] who verified that housing system, breed and age of hen affects the blood parameters with significant interaction between the factors. The birds reared in floor system presented more variation on metabolism according to dietary CP compared to birds reared in cage system. This may indicate a different demand of protein by birds in relation to housing system. Likewise, the stocking density of birds differed according to the rearing system and may contributed to differences in bird’s metabolism observed.

Birds reared in the cage system and fed diets with 160 and 180 g/kg CP presented greater levels of uric acid. This response was expected because uric acid is the method of excretion of excess of dietary nitrogen. Birds reared on the floor and fed with 180 g/kg CP produced more than 200% more uric acid than those fed with 140 g/kg CP. The excretion of uric acid increases when the diet contains an excess of CP. Besides the function of nitrogen excretion, uric acid had been related to one of the most important antioxidants in birds and is linked to their longevity [25].

The relative weight of small intestine and oviduct of laying hens reared in floor pens was greater than hens reared in cages. These results are in agreement with Yang et al [26] who reported that the weight of proventriculus, gizzard, liver, spleen, duodenum, jejunum, ileum, cecum, and rectum of free-range birds was higher than those of cagereared. Some researchers determined that organ weights were heavier in hens ranged on pasture and related that the pasture intake increased digestive organ weight [27], and fiber intake [28]. In the present study the birds were reared in floor with rice hulls as a litter, however, they did not had access to the pasture.

On the other hand, it is noticed that the floor system resulted in lower relative liver and pancreas weights of birds. These organs are responsible for enzyme production among other functions and are affected principally by diets. The liver weight has been related to stress response in laying hens [19]. Some authors verified that dry weight, moisture, protein, fat, carbohydrate, and ash contents of liver were all increased in laying hens under stress mediated by continuous infusion of ACTH [19]. The higher liver weight can indicate a metabolic disorder called fatty liver haemorrhagic syndrome (FLHS). Factors associated with husbandry practices in different production systems, such as restricted movement, increased production and temperature variations, influence hepatic lipid metabolism and predispose hens to FLHS [29].

The oviduct length, the percentage of abdominal fat, the relative crest and dewlap weights were not affected by the rearing system. These characteristics are related to reproductive performance of laying hens. Because of the stress response, the weight of the oviduct of laying hens can be reduced [19,30]. According to Wang et al [31] the elevated corticosterone levels in response to stressors may be associated with suppressed reproduction in laying hens via a possible perturbation of the hypothalamic-pituitary-gonadal axis. Therefore, based in reproductive characteristics, the birds did not present symptoms of stress according to the rearing system studied.

The reduction of dietary CP with amino acids supplementation aims to minimize the pollution with nitrogen products from poultry excretion [32]. Furthermore, the reduction of CP with amino acid supplementation improves the hens’ egg production, the small intestine villus height and promoting beneficial effects of faecal microflora [33].

In the present study, the small intestine length and relative weight of small intestine were affected by dietary CP. Both low and greater levels of CP negatively affected the intestine development. The other parameters evaluated were not affected by the dietary CP as an isolated factor, indicating that the reduction of dietary CP until 140 g/kg with amino acids supplementation can be used as a criterion for diet formulations.

Significant interactions were observed between protein level and raising system for some parameters studied (relative weights of gizzard crest and dewlap and for serum protein, uric acid, and total cholesterol content). The relative gizzard weight of birds reared in cage system was not affected by CP but as the dietetic CP content increased in birds reared in floor system there was a decrease of relative gizzard weight. The gizzard plays an important function in digestive process of diets, so according to Svihus et al [34] there are a renewed interest in nutritional effects in diets that can stimulate the development and function of the gizzard. Freitas et al [28] concluded that the neutral detergent fiber reduced gizzard weight of hens. In the present study, the reduction of CP was beneficial to gizzard development, however, more studies are required to elucidate these findings.

The reduction of CP until 140 g/kg CP and 150 g/kg to laying hens reared in cage and floor system, respectively, resulted in the highest relative crest weight. Birds on cage system showed greater relative dewlap weight when fed with 140 and 160 g/kg CP. Birds on floor system showed greater relative dewlap weight when fed diets with 160 and 180 g/kg of CP. These sexual characteristics of laying hens could be associated with productive performance and requires further investigation.

The diets with 140 g/kg CP resulted in lower serum protein and lower total cholesterol content in birds reared in floor system, indicating a possible CP deficiency. In contrast, birds reared in cage system showed no effect of CP on serum protein and total cholesterol. Some factors are related to cholesterol metabolism in hen, as different levels of methionine throughout the day [35] and fiber intake [36], however the hypocholesterolemic effect associated to the protein is unclear.

## Figures and Tables

**Table 1:** Composition of experimental diets

Items	140 CP (g/kg)	150 CP (g/kg)	160 CP (g/kg)	180 CP (g/kg)
Ingredients (g/kg)				
Corn grain	687.8	655.8	679.7	612.9
Soybean meal (45% crude protein)	179.1	206.2	134.8	188.7
Corn gluten meal (60% crude protein)	-	2.7	68.5	74.4
Soybean oil	16.2	21.2	2.3	13.2
Dicalcium phosphate	10.5	10.3	10.4	9.9
Limestone	91.8	91.8	92.0	92.0
Common salt	4.7	4.7	4.8	4.8
DL-methionine	2.9	2.7	1.8	1.2
Lysine HCL	2.1	1.2	2.9	1.1
L-valine	1.4	0.9	0.3	-
L-threonine	1.3	0.7	0.5	-
L-tryptophan	0.3	0.2	0.4	0.1
L-isoleucine	0.5	00.0	00.0	00.0
Vitamin supplement^1)^	1.0	1.0	1.0	1.0
Mineral supplement	0.5	0.5	0.5	0.5
BHT	0.1	0.1	0.1	0.1
Total	1,000.0	1,000.0	1,000.0	1,000.0
Calculated nutritional values				
Metabolizable energy (Mcal/kg)	2,900	2,900	2,900	2,900
Crude protein (g/kg)	140.0	150.0	160.0	180.0
Digestible lysine (g/kg)	7.5	7.5	7.5	7.5
Digestible methionine+cystine (g/kg)	6.9	6.9	6.9	6.9
Digestible methionine (g/kg)	4.8	4.7	4.5	4.2
Digestible valine (g/kg)	7.1	7.2	7.2	7.7
Digestible threonine ((g/kg)	5.7	5.7	5.7	6.0
Digestible tryptophan (g/kg)	1.7	1.7	1.7	1.7
Digestible isoleucine (g/kg)	5.7	5.7	6.0	7.0
Digestible arginine (g/kg)	8.1	8.9	8.0	9.5
Digestible phenylalanine (g/kg)	6.3	6.9	8.0	9.1
Glycine+serine (g/kg)	13.2	14.3	14.5	16.7
Digestible histidine (g/kg)	3.6	3.8	3.9	4.4
Digestible leucine (g/kg)	12.4	13.2	17.9	19.6
Linoleic acid (g/kg)	23.5	25.8	16.5	21.7
Calcium (g/kg)	38.5	38.5	38.5	38.5
Available phosphorus (g/kg)	2.8	2.8	2.8	2.8
Sodium (g/kg)	2.1	2.1	2.1	2.1

CP, crude protein.

1) Supplementation levels (amount per kg of product): 10.000 IU of Vit A; 2.000 IU of Vit D_3_; 1.833 mg of Vit E; 2 mg of Vit B_1_; 1.000 mg of vit B_2_; 3 mg of Vit B_6_; 0.015 mg vit B_12_; 12 mg of pantothenic acid; 3 mg of Vit K_3_; 1 mg of folic acid; 0.25 mg of selenium. 33.333 mg of manganese; 6.567 mg of iron; 2.667 mg of copper; 250 mg of iodine; 26.667 mg of Zinc; 6.000 mg of Niacin; 70,000 mg of choline; 680 mg of ethoxyquin; 8.333 mg of halquinol.

**Table 2:** The relative weight of digestive organs (g/100 g of body weight) and small intestine length (cm) of laying hens reared different systems and fed diets with different levels of crude protein

Rearing system	Crude protein	Small intestine length (cm)	Relative weight of small intestine (%)	Relative liver weight (%)	Relative gizzard weight (%)	Relative pancreas weight (%)
Interaction effects						
Cage	140	115.1	4.366	2.584	1.634^a^	0.216
Cage	150	134.5	3.814	2.442	1.502^a^	0.220
Cage	160	132.3	4.310	2.566	1.664^a^	0.228
Cage	180	123.8	3.946	2.392	1.424^a^	0.200
Floor	140	131.9	4.564	2.158	1.978^a^	0.186
Floor	150	140.4	4.198	2.058	1.870^a^	0.206
Floor	160	128.4	4.478	2.166	1.616^b^	0.194
Floor	180	122.8	4.174	2.100	1.504^b^	0.180
Main effects						
Cage		126.42	4.109^b^	2.4960^a^	1.556	0.2160^a^
Floor		130.87	4.353^a^	2.1205^b^	1.742	0.1915^b^
	140	123.50^b^	4.465^a^	2.371	1.806	0.201
	150	137.45^a^	4.006^b^	2.250	1.686	0.213
	160	130.35^a^	4.394^a^	2.366	1.640	0.211
	180	123.30^b^	4.060^b^	2.246	1.464	0.190
SEM		2.069	0.064	0.053	0.036	0.006
CV (%)		8.04	7.61	11.42	11.00	13.49
		------------------------------------------------------------------ ANOVA (p-value) ------------------------------------------------------				
S		0.18	0.02	<0.001	<0.001	<0.001
CP		0.01	0.01	0.56	<0.001	0.24
S×CP		0.14	0.88	0.95	0.04	0.84

SEM, standard error of the mean; ANOVA, analysis of variance; CV, coefficient of variation; S, rearing system; CP, crude protein.

a,b Means within a column-subgroup with no common superscript letters are significantly different at p<0.05 by Scott Knott test.

**Table 3:** The relative weight of reproductive organs (g/100 g of body weight) and oviduct length (cm) of laying hens reared different systems and fed diets with different levels of crude protein

Rearing system	Crude protein	Oviduct length (cm)	Relative oviduct weight (%)	Relative weight of abdominal fat (%)	Relative crest weight (%)	Relative weight of dewlap (%)
Interaction effects						
Cage	140	61.6	3.530	2.418	0.3000^a^	0.1867^a^
Cage	150	60.4	3.286	2.050	0.2275^b^	0.1520^b^
Cage	160	59.4	3.356	2.236	0.2480^b^	0.1760^a^
Cage	180	58.9	3.146	1.902	0.1900^b^	0.1560^b^
Floor	140	60.1	4.084	1.862	0.2340^a^	0.1425^a^
Floor	150	54.1	3.980	1.968	0.2560^a^	0.1540^a^
Floor	160	53.8	4.378	2.314	0.2125^b^	0.1140^b^
Floor	180	60.1	4.270	2.168	0.1850^b^	0.1060^b^
Main effects						
Cage		60.075	3.3295^b^	2.1515	0.2414	0.1677
Floor		57.025	4.1780^a^	2.0780	0.2219	0.1291
	140	60.85	3.807	2.140	0.2670	0.1646
	150	57.25	3.633	2.009	0.2418	0.1530
	160	56.60	3.867	2.275	0.2302	0.1450
	180	59.50	3.708	2.035	0.1875	0.1310
SEM		1.252	0.085	0.086	0.007	0.013
CV (%)		10.69	11.35	20.32	15.41	13.55
		------------------------------------------------------------------ ANOVA (p-value) --------------------------------------------------------------				
S		0.13	<0.001	0.59	0.11	<0.001
CP		0.41	0.62	0.51	<0.001	0.01
S×CP		0.50	0.41	0.19	0.05	<0.001

SEM, standard error of the mean; CV, coefficient of variation; ANOVA, analysis of variance; S, rearing system; CP, crude protein.

a,b Means within a column-subgroup with no common superscript letters are significantly different at p<0.05 by Scott Knott test.

**Table 4:** The blood parameters of laying hens reared different systems and fed diets with different levels of crude protein

Rearing system	Crude protein	Serum protein (g/dL)	Albumin (g/dL)	Uric acid (mg/dL)	Total cholesterol (mg/dL)
Interaction effects					
Cage	140	5.010^a^	2.252	2.630^b^	123.10^a^
Cage	150	4.336^a^	2.184	2.932^b^	105.88^a^
Cage	160	4.218^a^	1.968	3.980^a^	108.32^a^
Cage	180	4.460^a^	2.050	3.780^a^	114.52^a^
Floor	140	3.928^b^	1.916	2.782^c^	86.44^c^
Floor	150	4.738^a^	2.234	2.688^c^	113.98^b^
Floor	160	5.054^a^	2.324	4.110^b^	123.80^b^
Floor	180	4.948^a^	2.336	6.378^a^	151.65^a^
Main effects					
Cage		4.506	2.1135	3.3305	112.95
Floor		4.667	2.2025	3.9895	118.96
	140	4.469	2.084	2.706	104.770
	150	4.537	2.209	2.810	109.930
	160	4.636	2.146	4.045	116.060
	180	4.704	2.084	5.079	133.085
SEM		0.132	0.069	0.145	4.612
CV(%)		14.41	15.98	20.68	19.89
		---------------------------------------------------------------- ANOVA (p-value) ---------------------------------------------------------			
S		0.45	0.42	<0.001	0.41
CP		0.86	0.85	<0.001	0.05
S×CP		0.01	0.12	<0.001	<0.001

SEM, standard error of the mean; CV, coefficient of variation; ANOVA, analysis of variance; S, rearing system; CP, crude protein.

a–c Means within a column-subgroup with no common superscript letters are significantly different at p<0.05 by Scott Knott test.

## References

[1] Casey-Trott TM, Guerin MT, Sandilands V, Torrey S, Widowski TM (2017). Rearing system affects prevalence of keel-bone damage in laying hens: a longitudinal study of four consecutive flocks. Poult Sci.

[2] Brantsæter M, Nordgreen J, Rodenburg TB, Tahamtani FM, Popova A, Janczak AM (2016). Exposure to increased environmental complexity during rearing reduces fearfulness and increases use of three-dimensional space in laying hens (Gallus gallus domesticus). Front Vet Sci.

[3] Netto DA, Lima HJD, Alves JR, Morais BC, Rosa MS, Bittencourt TM (2018). Production of laying hens in different rearing systems under hot weather. Acta Sci.

[4] Singh R, Cheng KM, Silversides FG (2009). Production performance and egg quality of four strains of laying hens kept in conventional cages and floor pens. Poult Sci.

[5] Radu-Rusu RM, Usturoi MG, Leahu A, Amariei S, Radu-Rusu CG, Vacaru-Opriş I (2014). Chemical features, cholesterol and energy content of table hen eggs from conventional and alternative farming systems. S Afr J Anim Sci.

[6] Galic A, Filipovic D, Janjecic Z (2019). Physical and mechanical characteristics of Hisex Brown hen eggs from three different housing systems. S Afr J Anim Sci.

[7] Heflin LE, Malheiros R, Anderson KE, Johnson LK, Raatz SK (2018). Mineral content of eggs differs with hen strain, age, and rearing environment. Poult Sci.

[8] Engel JM, Widowski TM, Tilbrook AJ, Butler KL, Hemsworth PH (2018). The effects of floor space and nest box access on the physiology and behavior of caged laying hens. Poult Sci.

[9] Bezerra RM, Costa FGP, Givisiez PEN, Goulart CC, Santos RA, Lima MR (2015). Glutamic acid supplementation on low protein diets for laying hens. Acta Sci.

[10] Englmaierová M, Tůmová E, Charvátová V, Skřivan M (2014). Effects of laying hens housing system on laying performance, egg quality characteristics, and egg microbial contamination. Czech J Anim Sci.

[11] de Almeida Brainer MM, Rabello CBV, dos Santos MJB (2015). Crude protein requirements of free-range laying hens. Anim Prod Sci.

[12] Tufarelli V, Baghban-Kanani P, Azimi-Youvalari S (2012). Effects of horsetail (Equisetum arvense) and spirulina (Spirulina platensis) dietary supplementation on laying hens productivity and oxidative status. Animals.

[13] Bozakova NA, Sotirov LK, Sasakova N, Lakticova KV (2015). Welfare improvement in laying hens during the hot period under a semi-open rearing system through dietary arginine and vitamin C supplementation. Bulg J Vet Med.

[14] Kang S, Da-Hye K, Lee S (2020). An acute, rather than progressive, increase in temperature-humidity index has severe effects on mortality in laying hens. Front Vet Sci.

[15] Gonzalez FHD, Scheffer JFS, Gonzalez FHD, Compos R (2003). Blood profile: clinical, metabolic and nutritional analysis tools. Anais do1º Simpósio de Patologia Clínica Veterinária da Regiao Sul do Brazil.

[16] De Vylder J, Dewulf J, Van Hoorebeke S (2011). Horizontal transmission of Salmonella Enteritidis in groups of experimentally infected laying hens housed in different housing systems. Poult Sci.

[17] Parisi MA, Northcutt JK, Smith DP, Steinberg EL, Dawson PL (2015). Microbiological contamination of shell eggs produced in conventional and free-range housing systems. Food Control.

[18] Rostagno HS, Albino LFT, Donzele JL, Viçosa MG (2011). Brazilian tables for poultry and swine.

[19] Mumma JO, Thaxton JP, Vizzier-Thaxton Y, Dodson WL (2006). Physiological stress in laying hens. Poult Sci.

[20] Basmacioğlu H, Ergül M (2005). Research on the factors affecting cholesterol content and some other characteristics of eggs in laying hens the effects of genotype and rearing system. Turk J Vet Anim Sci.

[21] Kraus A, Zita L, Krunt O, Härtlová H, Chmelíková E (2021). Determination of selected biochemical parameters in blood serum and egg quality of Czech and Slovak native hens depending on the housing system and hen age. Poult Sci.

[22] Silva PRL, Freitas Neto OC, Laurentiz AC, Junqueira OM, Fagliari JJ (2007). Blood serum components and serum protein test of Hybro-PG broilers of different ages. Braz J Poult Sci.

[23] Lloyd S, Gibson JS (2006). Haematology and biochemistry in healthy young pheasants and red-legged partridges and effects of spironucleosis on these parameters. Avian Pathol.

[24] Alkhalf A, Alhaj M, Al-Homidan I (2010). Influence of probiotic supplementation on blood parameters and growth performance in broiler chickens. Saudi J Biol Sci.

[25] Simoyi MF, Van Dyke K, Klandorf H (2002). Manipulation of plasma uric acid in broiler chicks and its effect on leukocyte oxidative activity. Am J Physiol Regul Integr Comp Physiol.

[26] Yang HM, Yang Z, Wang W (2014). Effects of different housing systems on visceral organs, serum biochemical proportions, immune performance and egg quality of laying hens. Eur Poult Sci.

[27] Iqbal Z, Roberts J, Perez-Maldonado RA, Goodarzi Boroojeni F, Swick RA, Ruhnke I (2018). Pasture, multi-enzymes, benzoic acid and essential oils positively influence performance, intestinal organ weight and egg quality in free-range laying hens. Br Poult Sci.

[28] Freitas ER, Braz NM, Watanabe PH, Cruz CEB, Nascimento GAJ, Bezerra RM (2014). Fiber level for laying hens during the growing phase. Ciênc Agrotec.

[29] Shini A, Shini S, Bryden WL (2019). Fatty liver haemorrhagic syndrome occurrence in laying hens: impact of production system. Avian Pathol.

[30] Attia YA, Abd El AEHE, Abedalla AA (2016). Laying performance, digestibility and plasma hormones in laying hens exposed to chronic heat stress as affected by betaine, vitamin C, and/or vitamin E supplementation. SpringerPlus.

[31] Wang XJ, Liu L, Zhao JP, Jiao HC, Lin H (2017). Stress impairs the reproduction of laying hens: an involvement of energy. World’s Poult Sci J.

[32] Alagawany M, Abd El-Hack ME, Arif M, Ashour EA (2016). Individual and combined effects of crude protein, methionine, and probiotic levels on laying hen productive performance and nitrogen pollution in the manure. Environ Sci Pollut Res Int.

[33] Tenesa M, Loh TC, Foo HL, Samsudin AA, Mohamad R, Raha AR (2016). Effects of feeding different levels of low crude protein diets with different levels of amino acids supplementation on layer hen performance. Pertanika J Trop Agric Sci.

[34] Svihus B (2011). The gizzard: function, influence of diet structure and effects on nutrient availability. Worlds Poult Sci J.

[35] Liu Y, Wan D, Zhou X (2019). Effects of dynamic feeding low- and high-methionine diets on the variation of glucose and lipid metabolism-related genes in the liver of laying hens. Poult Sci.

[36] Laudadio V, Ceci E, Lastella NMB, Tufarelli V (2014). Effect of feeding low-fiber fraction of air-classified sunflower (Helianthus annus L.) meal on laying hen productive performance and egg yolk cholesterol. Poult Sci.

